# Addressing obstacles to the inclusion of palliative care in humanitarian health projects: a qualitative study of humanitarian health professionals’ and policy makers’ perceptions

**DOI:** 10.1186/s13031-020-00314-9

**Published:** 2020-10-28

**Authors:** Matthew Hunt, Elysée Nouvet, Ani Chénier, Gautham Krishnaraj, Carrie Bernard, Kevin Bezanson, Sonya de Laat, Lisa Schwartz

**Affiliations:** 1 School of Physical and Occupational Therapy, McGill University; Researcher, Centre for Interdisciplinary Research on Rehabilitation, 3654 Prom Sir William Osler, Montreal, QC H3G 1Y5 Canada; 2 School of Health Studies, Western University, HSB 339 1151 Richmond St, London, ON N6A 5B9 Canada; 3 Humanitarian Health Ethics Research Group, Western University, School of Health Studies, Western University, School of Health Studies, HSB 339, 1151 Richmond St, London, ON N6A 5B9 Canada; 4 Humanitarian Health Ethics Research Group, McMaster University, CRL Building, 1280 Main Street West, Hamilton, Ontario L8S 4K1 Canada; 5 Department of Community and Family Medicine, University of Toronto, 500 University Ave, Toronto, ON M5G 1V7 Canada; 6 Department of Family Medicine, McMaster University 100 Main Street West, 6th Floor, Hamilton, ON L8P 1H6 Canada; 7 Northern Ontario School of Medicine, Lakehead University, Thunder Bay Regional Health Sciences Centre, 980 Oliver Rd, Thunder Bay, Ontario P7B 6V4 Canada; 8 Global Health, McMaster University, 1280 Main Street West, MDCL 3500, Hamilton, ON L8S 4K1 Canada; 9 Department of Health Research Methods & Impact, McMaster University, CRL Building, 1280 Main Street West, Hamilton, Ontario L8S 4K1 Canada

**Keywords:** Armed conflict, Disasters, Ebola virus disease, end of life, Ethics, humanitarian action, non-governmental organizations, palliative care, Public health emergencies

## Abstract

**Background:**

Humanitarian non-governmental organizations provide assistance to communities affected by war, disaster and epidemic. A primary focus of healthcare provision by these organizations is saving lives; however, curative care will not be sufficient, appropriate, or available for some patients. In these instances, palliative care approaches to ease suffering and promote dignity are needed. Though several recent initiatives have increased the probability of palliative care being included in humanitarian healthcare response, palliative care remains minimally integrated in humanitarian health projects.

**Methods:**

We conducted a qualitative study using interpretive description methodology to investigate humanitarian policy-makers’ and health care professionals’ experiences and perceptions of palliative care during humanitarian crises. In this article, we report on the analysis of in-depth interviews with 24 participants related to their perceptions of obstacles to providing palliative care in humanitarian crises, and opportunities for overcoming these obstacles. Among the participants, 23 had experience as humanitarian health professionals, and 12 had experience with policy development and organizational decision-making.

**Results:**

Participants discussed various obstacles to the provision of palliative care in humanitarian crises. More prominent obstacles were linked to the life-saving ethos of humanitarian organizations, priority setting of scarce resources, institutional and donor funding, availability of guidance and expertise in palliative care, access to medication, and cultural specificity around death and dying. Less prominent obstacles related to continuity of care after project closure, equity, security concerns, and terminology.

**Conclusion:**

Opportunities exist for overcoming the obstacles to providing palliative care in humanitarian crises. Doing so is necessary to ensure that humanitarian healthcare can fulfill its objectives not only of saving lives, but also of alleviating suffering and promoting dignity of individuals who are ill or injured during a humanitarian crises, including persons who are dying or likely to die.

## Background

The toll in suffering and loss of life due to humanitarian crises – including wars, disasters and epidemics – is staggering. In 2018, an estimated 206 million people in 81 countries required humanitarian assistance and over 70 million people were forced to flee their homes due to crises [1]. In some instances, local communities and national organizations have the capacity to address the needs of affected populations. However, in other settings, especially during large scale crises, or crises occurring in countries with limited resources, a range of international organizations and entities provide assistance, including non-governmental organizations (NGOs) and intergovernmental organizations. This article focuses on the work of these organizations in providing healthcare for communities affected by crises and, particularly, the obstacles and opportunities for these organizations to provide palliative care in humanitarian contexts. As defined by the World Health Organization (WHO), “[p] alliative care is an approach that improves the quality of life of patients and their families facing problems associated with life-threatening illness, through the prevention and relief of suffering by means of early identification and impeccable assessment and treatment of pain and other problems, physical, psychosocial and spiritual.” [2] More recently, the Lancet Commission on Palliative Care has described palliative care as “an essential component of comprehensive care for persons with complex chronic or acute, life-threatening, or life- limiting health conditions” [[3], p 1400].

The goals of humanitarian action are threefold: “to save lives, alleviate suffering and maintain human dignity.” [4] While these three objectives are oft repeated, the roles they play in guiding priorities and practices in humanitarian health care are rarely equal. Recently, scholars and humanitarian practitioners have highlighted this imbalance, noting that efforts to address suffering and uphold the dignity of individuals, especially for those whose lives cannot be saved during a humanitarian crisis, have received limited attention in the sector [5–7]. The international response to the 2014–15 Ebola epidemic in West Africa made this reality all the more evident [8], as humanitarian healthcare teams that usually focused on lifesaving strategies struggled to face the reality that supportive care was the main treatment available.

The descriptions in the following three boxes exemplify sentinel scenarios that guided the research that is the focus of this article. They draw attention to situations in which a patient is being cared for by a humanitarian healthcare team, and for whom alleviation of suffering and respect for dignity are desperately needed. The families of these patients also need support and guidance.

**Table:** 

Scenario 1: Mass casualty triage.
Following an earthquake resulting in hundreds of deaths and severe damage to local infrastructure, the wounded are presenting to an emergency field hospital. Medical staff are triaging people to different areas for immediate life-saving care, less serious injuries, and those whose injuries are too severe to survive and are deemed unsalvageable. One such young man has a severe crush injury. He is confused and agitated, complaining of thirst, and moaning in pain.

**Table:** 

Scenario 2: End-stage disease.
An NGO is responsible for care provision in a refugee camp bordering a country with ongoing and evolving civil war. A woman who was forced to flee two weeks ago arrives with her teenage son. Prior to fleeing she was receiving hemodialysis for end-stage renal disease. The camp does not have access to dialysis, and the physician assessing her expects she will deteriorate and die within the coming few weeks with the limited available care.

**Table:** 

Scenario 3: Incurable condition.
An Ebola Treatment Center has been established to care for patients at a time when the case fatality rate approaches 60%. All care is provided in full Personal Protective Equipment, minimizing time and contact with patients. A woman from an outlying area has been admitted to the Center. Family members are not allowed to enter the Center due to concerns of contagion and she has had no contact with family since her arrival. Despite supportive treatment she is deteriorating rapidly with a dire prognosis. While experiencing ongoing diarrhea and vomiting, she is delirious, distressed, and calling out for loved ones.

As noted above, the need for humanitarian organizations to integrate actions to address pain and suffering is increasingly recognized [9]. Important guidelines have been published in the last two years [10–12]. Continuing to extend humanitarian organizations’ capacities to address these goals – including integrating palliative care approaches more widely and indeed beyond those dying or likely to die – requires scrutiny of lingering obstacles to providing these forms of care, and the development and implementation of strategies to overcome barriers. Findings from this study help to clarify barriers and opportunities to address them.

## Methods

We undertook an exploratory qualitative study from within a constructivist paradigm [13] and based on interpretive description methodology [14] to investigate humanitarian health care professionals’ and policy-makers’ experiences and perceptions of palliative care during humanitarian crises. Interpretive description originated in nursing sciences, with the aim of developing knowledge to guide practice in applied health disciplines. An interpretive description aims to better understand a phenomenon by illuminating its “characteristics, patterns and structure” while being attentive to variation and divergence, and to understand the meanings that individuals bring to their experiences [15]. This article presents an analysis of how our participants perceived obstacles to addressing palliative care in humanitarian crises, and opportunities for overcoming these obstacles. In a separate article, we present an analysis of the participants’ moral experiences, that is situations in which they felt that values that they deemed to be important were being realized or thwarted, in providing care for patients who were dying or likely to die in a humanitarian context [16]. We note that the stories discussed by the participants in this study were heavily focused on situations when severely ill or injured individuals were dying or likely to die, and we acknowledge that palliative care is relevant to a broader array of health situations [3]. This set of 24 interviews is part of a larger program of research which included an international survey, and a set of four studies in refugee camps in Jordan and Rwanda, about care in Ebola Treatment Centers in Guinea, and focused on palliative care during or following natural disasters [17].

### Recruitment

We used four strategies to recruit humanitarian health professionals and policy-makers. We distributed information about this study through our research group’s Twitter and Facebook accounts. We then circulated this information within our professional networks (11 participants recruited). The third means of recruitment was through a survey that we conducted on palliative care practices and policies in humanitarian organizations. At the completion of the survey, respondents were invited to provide their contact information if they were interested to participate in an in-depth interview (10 participants). The final strategy was snowballing: interview participants were invited to suggest other potential participants (3 participants). As recruitment progressed, we sought to recruit participants from diverse organizations, with experience in a wide range of humanitarian crises, having different professional roles and backgrounds, and offering varied perspectives on the phenomenon of palliative care in humanitarian crises.

### Participants

We interviewed 24 participants. Twelve participants identified as having a policy-making role in a humanitarian setting or organization. These individuals occupied diverse positions, ranging from country coordinators for a NGO, to individuals working at the international headquarters of aid agencies, to health professionals involved in writing clinical guidelines for humanitarian settings. These individuals had prior experience working as health professionals, with the exception being a senior manager within a humanitarian organization. The second group of participants consisted of 12 health professionals with experience providing healthcare as part of an international humanitarian NGO. In total, the participants had experience with 19 different organizations involved in healthcare delivery during humanitarian crises, though most frequently they were affiliated with larger international organizations based in Europe or North America. The number of participants who had worked with each organization ranged from one to eight (of these eight, five had experience with at least one other organization). The sample included 12 men and 12 women. Twenty participants were from high income countries and four from low- or middle-income countries.

### Interviews

Semi-structured in-depth interviews were conducted between November 2016 and May 2017, by Skype or telephone, in English or French according to the preference of the participant. Interviews were audio recorded and transcribed verbatim. The participants were asked to share their understanding and experiences with palliative care needs in humanitarian crises, along with their perception of key challenges to the provision of this care, and ideas about whether or not, and how, these should be addressed. Interview guides were refined based on feedback from individuals with expertise in the areas of palliative care, humanitarian healthcare and qualitative research, and were tailored for participants with experience only as health professionals, and for participants who had experience as policy-makers. Example questions include: What, if any, training have you had related to palliative care in general, or palliative care in humanitarian crises? What is your experience/role in relation to the provision of palliative care in humanitarian crises? How do (es) the organization(s) with which you have worked approach the issue of palliative care? Based on your experience, what suggestions would you make to improve the care for patients and families needing palliative care in the context of humanitarian response? Interviews averaged 65 min in duration.

### Data analysis

Analysis was initiated as soon as transcripts became available. First-level inductive coding was conducted by two members of the research team using NVivo software (there were separate coders for the policy-maker (GK) and health professional (AC) interviews). Codes were developed in English across both French and English transcripts in order to establish a consistent analytic structure. Coding used constant comparative techniques and involved asking the questions “What is going on here?” and “What is this about?” while reading sections of the text. A second team member (MH) independently coded sections of four transcripts (two for policy-makers and two for health professionals). The preliminary coding structure was revised through comparison of the coded transcript excerpts, and through feedback received from three other members of the research team after they had reviewed additional interview transcripts (EN, KB, CB). The analysis presented in this article resulted from a process of reviewing the coded transcripts asking the questions: “How does this relate to obstacles to providing palliative care?” and “How does this relate to opportunities to overcome obstacles to providing palliative care?” Rather than presenting findings separate from discussion, these two elements are merged in the presentation of our analysis below.

## Results and discussion

Diverse features of humanitarian crises, humanitarian organizations and humanitarian healthcare, as well as broader political and legal structures, contribute to obstacles to further integrate palliative care approaches in humanitarian settings. Through our interviews with humanitarian health professionals and policy-makers, we identified ten obstacles. In what follows, we begin by discussing the six more prominent obstacles, sequenced to reflect a logical flow of ideas. As well as describing the obstacle, we outline and discuss opportunities identified by participants for addressing it. We then summarize the four less prominent obstacles. Several participants offered cautions regarding advocacy for palliative care approaches in humanitarian action. We conclude with a summary of those cautions.

### Ethos: primacy of lifesaving in humanitarian action

A key obstacle identified by participants related to the ethos of humanitarian action: the primacy of life-saving over other objectives, including the alleviation of suffering and promotion of dignity. All participants endorsed the idea that saving lives is a primary commitment of humanitarian action. However, they also discussed ways that the overarching focus on life saving can squeeze out the opportunity for other sorts of actions, including care aiming to ease suffering for persons with life threatening conditions and those who are dying. A participant described how, in the context of the Ebola outbreak in West Africa, *“I think it becomes a lot of, you know, trying to avert mortality so much that we sort of … not avoid, but just forget to think about the … sort of human dignity aspect [of] … dying during an outbreak.”*

The context of humanitarian action may also contribute to the perception that efforts beyond lifesaving are not feasible. Humanitarian healthcare projects are implemented in dynamic, chaotic contexts where resources are scarce and needs are both elevated and widespread. In discussing their experiences after a major earthquake had occurred, a participant expressed that “*most times in humanitarian emergencies, I think the biggest problem is actually convincing people to also think of palliation because with emergencies, with the rush, with the pressure of people everywhere, it’s usually very hard.*” In such situations, health professionals may feel that they do not have time to provide palliative care, and several participants described that some of their colleagues viewed palliative care as a luxury rather than an integral component of health care in crisis settings.

Increasingly, humanitarian organizations and practitioners are discussing palliative and end-of-life care, while acknowledging the constraints that exist during crises [5, 18]. Participants described two developments contributing to this shift. The first was the response to the 2014–15 West African Ebola epidemic in which supportive care played a large role due to the lack of curative options, yet was implemented in a context where isolation and infection control protocols constrained such efforts. The second is the increased prevalence of individuals with end stage chronic diseases receiving humanitarian healthcare in countries, such as Syria, which had a well-functioning health system prior to the onset of war. A physican noted how the increased prevalence of chronic conditions in many humanitarian projects requires reassessment of previous views on caring for dying patients:

*I think it’s part of the evolution of the humanitarian … from life-saving interventions from the beginning of very basic interventions for life-saving, now it’s quite more complex nowadays. I mean there are a lot of specialized projects. We are talking about projects of just chronic diseases. I mean with chronic diseases, they are not, it’s not that direct life-saving intervention. I think it’s clear that [palliative care in humanitarian crisis] has been neglected until now. We have neglected it because it – it’s very easy to focus, forget about it and focus [on] that we have to save lives. But it’s true that if you look to the statistics and everything there’s a lot of patients [who] will not survive, and ethically and medically they need this support really.*

Our participants did not question the importance of lifesaving action. Rather, they consistently argued that the humanitarian approach should make room for addressing suffering and dignity *alongside* efforts to prevent mortality. On the whole, participants supported more integrated approaches that did not separate curative and palliative care, but sought to integrate them. Organizations can play a central role in this process by emphasizing that compassionate care for the dying is an integral part of their work, thus acknowledging that these patients exist and that responding to their suffering and promoting their dignity are important aspects of humanitarian healthcare. As discussions expand within organizations (such as through processes to develop training or policy), the ethos of humanitarian healthcare is likely to shift and become more inclusive of palliative care.

### Priority setting: allocating limited humanitarian resources

While the primacy of life-saving may lead to palliative care needs being less visible, a related obstacle pertains to prioritizing limited humanitarian health care resources even when palliative care needs are recognized as important. A participant expressed this objection as a rhetorical question: *“how is it possible to ensure palliative care in the absence of basic curative care?”* This question goes beyond allocation of scarce resources, and points to the global inequalities and structures that shape possibilities of accessing resources. A physician who had participated in humanitarian projects in several armed conflicts reflected: *“I think the added wrinkle in humanitarian contexts is that sometimes there are patients who wouldn’t be palliative in another context, but because they become so, and that can be difficult.”* And yet, the need to prioritize is an urgent reality in humanitarian settings. The prioritization of life-saving goals in a humanitarian project was described by another participant. He reported his experience in a project where many patients’ lives were at risk and resources were extremely limited: “*So these patients [who were dying] were put aside and basically, literally nobody would care for their needs. Because I was under enormous pressure to help those who had a chance for cure.”*

During humanitarian crisis events, questions of resource allocation arise at multiple levels. Participants commonly described time and effort of healthcare teams as routinely requiring prioritization in the field. Less often, allocation choices were called for to determine the best use of material resources such as medications or beds. A participant who worked with one of the largest humanitarian NGOs noted that, in a crisis context, there are *“strong limitations because of money, materials, and most often, it’s human skill, human resources.”*

Perhaps the most striking stories related to human and material resource prioritization were those involving triage decisions. In several scenarios described in detail by participants, little or no care was provided to those persons who were placed in the category of ‘expectant’ or given a ‘black tag’ to denote that they were considered unsalvageable within a triage scheme. A participant described how *“for very long these ones … were not really properly assisted because the objective was life-saving.”* Palliative care may thus be seen as unjustified or categorized as “*a luxury*”– even if recognized as important – where resources are severely limited. This is particularly the case if providing pain relief or addressing suffering is perceived as possibly taking away, due to resource limitations, from the team’s ability to provide curative care to people whose lives might be saved. For example, a participant described a colleague who held that “*you don’t want to think about palliative care in emergency contexts”* because they felt it would take energy away from life-saving efforts.

Despite the undeniable need for triage, several opportunities were identified by participants to adapt or reframe practices in ways that would have implications for priority-setting. Participants described a range of palliative care interventions that were not expensive or resource-intensive, nor required expert care providers. Active listening, spiritual care, holding someone’s hand, sitting at their bedside, or keeping the patient’s lips moist were all identified as important ways to address suffering and demonstrate compassion. Important aspects of palliative care, it was thus proposed, can be provided by people who do not have specialized training. Participants recounted efforts to incorporate community workers and other lay caregivers allowing healthcare teams to provide better palliative care than the available resources would otherwise have allowed. In the experience of our participants, such efforts appear to be more common in chronic or protracted crisis situations, especially where it is not possible to admit patients into formal care settings. Some participants recounted working, informally, to equip patients’ family members with knowledge and skills that would help them care for their relatives. For example, a participant described helping a patient with advanced cancer by teaching her family members how to dress her wounds and do basic mobilization exercises. A few participants also described more formal efforts to integrate lay caregivers and volunteers into care plans, including by tasking them with providing comfort care for those persons categorized as unsalvageable through a triage protocol. Below, we describe further recommendations related to training and availability of resources which are also highly relevant to triage situations.

### Funding: public expectations and implications for fundraising

Participants reflected on how palliative care challenges the broader public discourse and popular imagination regarding humanitarianism. A participant described widespread perceptions of humanitarian healthcare as a *“heroic medicine that cures children and people*.” Several participants identified palliative care as fitting poorly with these public perceptions, a mismatch which could therefore interfere with humanitarian fundraising. A physician described her perspective of how individuals who provide funds to humanitarian agencies view the proper use of their funds: “*I think that people have this notion … that if you have funding and money and resources, then it should go completely and directly to saving a life. Which I don’t disagree with, but I also feel that we need to also ensure that everyone’s needs are met if possible.”* Several participants described this dynamic as contributing to reluctance within organizations to discuss palliative care. For example, a policy-maker reported that in fundraising *“what engages people well is ‘save a life’, it doesn’t sit as well with [the public] to ‘help someone die a dignified death.’”* However, it was also noted that when humanitarian organizations only emphasize life-saving in fundraising efforts, they risk further reinforcing these public perceptions, as well as perceptions within the humanitarian community.

A physician reported that “*I think sometimes that maybe palliative care is not seen now, nowadays, does not seem very fancy to the donors, or to the big public. Many times humanitarian organizations are supported because they are in the midst of places where you see the emergency, you know the rush, the lifesaving interventions more and more, more donors are focusing on this”* and *“maybe [it] doesn’t look so fancy, it’s more fancy to [have] surgeons of course who … are doing lifesaving interventions than to have a palliative care doctor or palliative care team who is taking care of those that are pining away, say they are going to die.”* This view of what is more attractive to donors may in turn drive organizational choices in terms of how they develop and implement their projects, for example preferring surgeons performing “*lifesaving interventions to hav [ing] a palliative care doctor or palliative care team who is taking care of those [who] are going to die.”* The perception that palliative care approaches are less easily marketed to the public or donors, and less likely to be valued by these actors, can thus be an obstacle to further integrating palliative care in humanitarian action.

Several avenues were proposed by participants for addressing these barriers. One proposal was to educate the public in donor countries about the importance of alleviating suffering and caring for the dying. A participant suggested that humanitarian organizations should be more forthcoming in their descriptions that these goals are also an integral part of their work during crises. One participant, who was a senior leader for a humanitarian organization based in Europe, reported that his organization intended to put these assumptions to the test. He described how the NGO with which he worked planned to focus a fundraising appeal on the alleviation of suffering for the dying, and planned to evaluate the fundraising campaign’s effectiveness relative to previous appeals in terms of the amount of funding that was raised.

In considering prevailing expectations that funds for humanitarian healthcare will and should be devoted to saving lives, it is important to consider how such expectations are normative rather than natural. In line with the idea of resisting a false dichotomy between curative and palliative care [5], organizations can consider emphasizing the contribution of these approaches across a continuum of care, and as approaches to be integrated in alignment with humanitarian goals and values.

### Guidance and expertise: access to consistent technical supports

Even when NGOs and teams are interested in incorporating palliative care into a humanitarian healthcare project, two additional gaps beyond scarce resources may prevent them from doing so: a lack of policies and guidelines, and/or insufficient expertise within the team. Participants described challenges related to the availability of actionable guidance, especially in the form of guidelines, protocols, policies and clinical standards. It is important to note that at the time of the interviews, several important sectoral guidelines had yet to be published, including the revised Sphere handbook [10], the WHO guide on integrating palliative care and symptom relief into responses to humanitarian emergencies and crises [12], and a field manual on palliative care in humanitarian crises [11]. Though a range of tools and resources were available, for example around how to communicate with dying patients and their families [19], they were rarely specific to humanitarian settings.

The absence of clear guidance and clinical protocols led to participants’ uncertainty for how to proceed, and contributed to inconsistencies within and between teams that participants described as problematic. A participant described how in a project she was involved in “*there was no guideline to go to, no book to go to, no protocol to go to.”* As a result, the team was unsure of how best to proceed. Developing policies and guidelines within organizations and across the humanitarian sector was seen as important for addressing this gap. For example, a participant recommended that NGOs should “*have at least this integration of the dimension of palliative care in all the protocols for triage and emergency situations.*” Several participants reported initiatives that were ongoing at the time of the interviews, including efforts to develop policies and procedures in the area of palliative care.

As well as sector-wide guidance, it is important that teams have specific guidance relevant to the nature and context of their work [20]. For example, healthcare teams require guidance for making decisions about allocating limited pain relief medications [21]. In some contexts, humanitarian healthcare may also require triage of palliative care efforts, attending first to those facing imminent risk of death or experiencing the most severe symptoms. Attention to contextual particularities, such as cultural beliefs and expectations about what should ideally be done and who should be ideally present as someone passes from life into death, represent important considerations for defining an ethics of palliative care in humanitarian practice. This is discussed in further detail below. Now that minimum standards and other guidelines are available [10–12], humanitarian organizations also need to engage in dialogue and discussion about their own practices and policies. This process may also lead to change in the ethos of these organizations.

Participants also noted that teams that lack members trained or experienced in palliative care approaches are less likely to integrate palliation in their clinical work. Training related to palliative care—both as a topic to be integrated across general training courses, or as the primary focus of specialized training activities—was viewed by many participants as crucial for augmenting the capacity to provide palliative care in humanitarian health projects. A participant linked training to increasing awareness of palliative care: “*the recognition of palliative care is one of the key pieces of a response to a disaster, so then the next piece is sort of teaching it, because people get so caught up in ‘How do I handle a crush injury?’ and … I think that … it should just [be] … part of the language that follows through.”* Training and preparation were thus described as key opportunities to address the obstacle of a lack of expertise. Training needs have also been highlighted in the literature [22]. However, some participants with expertise also recognized that other obstacles still can and do impede implementing this knowledge.

Many participants identified referral strategies as an approach for teams lacking capacity. This included physical referral to regional centers, particularly for diagnostic clarification, recognizing the financial and family burdens this can entail. The other proposed approach was to use telemedicine for consultation when more expertise was required. So, while training and guidelines can support basic palliative capacity, physical or telemedicine referral could be used to support more complex scenarios.

### Access to medication: availability of pain relief medications in humanitarian aid projects

A central preoccupation for participants was the challenge of accessing opioids in many countries where humanitarian crises occur. Barriers to access included legal restrictions, national and international regulations, perceptions, and supply chain issues. A participant described obstacles to using opioids: *“And first, the first cause is because opioids ... are absolutely not authorized in most of west African and central African countries. So even for us, if we want to use morphine, to get the authorization for importation is very, very, very, complicated.”* This barrier was also emphasized by a second participant: *“Another one of the main challenges I think is the availability of the drugs, the importation or the use of opioid drugs in many countries is forbidden or you don’t have access to it.”*

All participants saw access to opioids as necessary for effective pain relief: *“speaking about palliative care, if you have no morphine and no pain – nothing from this family … I can tell you that, already it’s a disaster. You cannot solve, almost, anything.”* Many emphasized, however, that palliative care approaches extended beyond provision of medication and that opioids were necessary but not sufficient for addressing the needs of patients in humanitarian crises. As a participant described, “*With ten medications, you can practice palliative medicine, pharmacologically speaking. But it is everything else. It is the communication, the relationship building, the dignity, it is all of those things*.*”* As discussed by participants, and consonant with the WHO definition [2], palliative care involves much more than using opioids to control pain. For example, palliative approaches include addressing some symptom issues such as nausea or delirium, spiritual needs, and caregiver issues, all areas of need that do not rely on the use of opioids. However, if patients are in unremitting pain or distress it is often extremely difficult to attend to other needs effectively.

Even where access to opioids was not legally restricted, their use was sometimes limited due to fears regarding addiction or perceptions among some health professionals and local communities that use of powerful analgesics were akin to *“giving up on the patient*.” These differing views, in participants’ accounts, sometimes led to disagreement between health professionals in terms of how they should respond to the patient’s needs. However, several participants identified that engaging these differences was essential to expanding access to effective pain management.

Limited access to pain medications, and especially opioids, is a significant barrier to more comprehensive palliative care approaches. It is clearly a complex problem with many contributing features. Improvements to humanitarian supply chains can lead to greater availability of opioids in some humanitarian projects. Elsewhere, issues related to legislation and regulations limiting opioids at national levels require ongoing advocacy. The humanitarian community has extensive experience advocating for essential medicines, including opioids. They have the opportunity to continue this process as a means to overcome barriers to access [3, 23].

### Cultural specificity: cultural understandings and practices related to death and dying

Participants emphasized the challenging dynamic of culture and palliative care in humanitarian crises, especially when healthcare is provided by international or national staff who come from a different community. Participants described their efforts to understand perceptions of patients and families, to manage issues such as taboos related to death, consider spiritual dimensions of dying and care for the dying, and to not act in ways that contravened local cultural norms. These cultural dimensions were highlighted by a physician who had worked in an Ebola Treatment Center: “*Palliation, I think, is huge, … largely because how death is managed and how death is … like a sort of social religious construct, is very different from … what some foreigners might be used to or [have] experienced.*” She continued*: “We were essentially taking away how death is … constructed and worked around, and … how families … think what is gonna happen to their loved ones and the first we’ve already taken them away, so they can’t see them die, they’re in the [Ebola Treatment Center] … they’re not there when their family member dies and that is a big thing already.”* Cultural specificity was seen as an obstacle in the sense that it creates a risk that humanitarian workers will unintentionally harm through failure to understand and integrate the nuances of culturally-appropriate norms and practices. Various opportunities for addressing this reality were also voiced.

Several participants emphasized the importance of working collaboratively with local communities. This view was expressed most strongly by a participant who is a physician from West Africa. He argued that palliative care should be “*more community-based, traditional healers, family members, community leaders. It should be of course with supervision from the government or international organizations, but I think it should be community-based. They should understand the language and stuff, the culture.*” Approaches to death and dying may also vary within humanitarian health care teams. A participant reported that *“there’s a cultural approach concerning the local staff that is clearly very*—*it’s very different, but also between expatriate staff.”*

Cultural meanings, practices and expectations associated with death and dying are varied. Failure to address cultural considerations do not make them go away. Doing so can have important negative impacts for the quality of care, and may harm trust and legitimacy of humanitarian healthcare. Humanitarian workers who come from outside a community are unlikely to have in-depth understanding of these considerations. Humility, willingness to learn and intentionality are needed, as well as self-awareness of one’s own cultural and personal associations with death and dying. In many situations, local health care providers and workers are part of the response, and can be engaged to guide the response in culturally and religiously appropriate directions. Additionally, local community leaders and interpreters need to be consulted for many aspects of care. It is critical to actively include communities in addressing issues of death and dying, and to do so in a manner that acknowledges potential inadequacies.

### Additional obstacles

Four other issues were raised in interviews as obstacles to the provision of palliative care in humanitarian crises, but were less prominent in the narratives of participants: equity between displaced populations and host communities, continuity of care, security, and terminology related to palliative care.

The first of these obstacles relates to concerns for equity when humanitarian aid is provided to refugees or other displaced populations, and the standard of care they receive exceeds that which is available to the host community via the local healthcare system. Though a general concern for all healthcare in these situations, it was discussed here in particular relation to the provision of palliative care and inequalities in what was available between refugee and host populations in a particular setting. The second related to concerns for ensuring adequate continuity of care. They were identified as an obstacle to initiating palliative care in some settings, especially for individuals whose health is failing and curative care is not deemed appropriate, but for whom death is not expected to be imminent. Such challenges were related to contexts where the pre-crisis health system lacked access to opioids and did not include palliative care, and therefore a return to stability may mean all palliative interventions would once again no longer be available. The third obstacle relates to concerns that providing palliative care could compromise security of a humanitarian healthcare team. Security may be compromised if local communities perceive humanitarians providing palliative care as having ‘given up’ on their patients or as being unwilling to provide life-saving care, situations which may undermine trust and lead to confrontation since “*we know that’s sometimes been — that [is] sometimes endangering our own teams.*” Finally, several participants also discussed how terminology can be an impediment to understanding and agreement in some organizational contexts. For example, a participant who was part of efforts to develop palliative care policies within her organization described the need to choose terminology that was perceived as more acceptable or neutral:

*“We speak about comfort care. That is the same for us [as] palliative care. But like I told you, it’s not known and poorly perceived. But comfort care, we try to say: ‘please, we cannot save, so we will discuss comfort.’”*

In this way, the term ‘palliative care’ itself might be seen as an obstacle to incorporating the treatment approaches that it represents. Careful attention is needed to how terms are understood and the role this plays in the acceptance of palliative care within different organizations and teams, or how it is understood by funders as being part of humanitarian healthcare.

A summary of all obstacles discussed by participants, and opportunities to address these, can be found in Table 1.

**Table 1:** A summary of obstacles and opportunities to address them

Obstacle	Summary	Opportunities to address it
Ethos	Primacy of life saving efforts in humanitarian action deflects attention from objectives of alleviating suffering and promoting dignity	● Increased reflection and engagement in humanitarian organizations ● Emphasizing all three goals of humanitarian action ● Accepting potential for integration of curative and palliative approaches ● Raise awareness of the unaddressed health-related suffering
Priority Setting	In situations of crisis where resources are scarce and needs are high, it may be difficult to justify directing resources to palliative care	● Emphasize that most palliative care interventions are not costly ● Partner with local community and lay caregivers ● Question when care being provided may be futile or unduly burdensome ● Ensure suffering and dignity are addressed for all patients as a matter of equity ● Prioritize those resources dedicated to palliative care to address needs of patients with most imminent and severe needs
Funding	Palliative care is unlikely to be effective for garnering funding from the public or large donors, a perception which may lead to not including it in programs	● Challenge perceptions of humanitarians as ‘heroic life-savers’ as it problematically narrows the scope of humanitarian action ● Test the assumptions that palliative care efforts would not be seen favorably by donors ● Learn from successful examples within and beyond the humanitarian sector (e.g hospice movement) ● Identify relevant accountability metrics for palliative care
Guidance and expertise	There are few organizational polices and clinical standards related to palliative care in humanitarian settings, and few organizations have developed expertise or implemented training in this area	● Develop policies, standards and clinical guidelines, and training for palliative care in humanitarian aid organizations ● Share resources among organizations ● Identify health professionals with palliative care expertise who can act as resources for the organization, and real time supports for teams
Access to medications	It is very difficult to access pain medications, especially opioids, in many countries due to legal restrictions, logistical issues, and misperceptions.	● Advocate for standard access to opioids and other pain and symptom medications, especially removal of legal barriers ● Plan and integrate medications into medical supply chain and logistics ● Address misperceptions regarding opioids
Cultural specificity	Humanitarian organizations and their staff coming from other settings will have difficulty accessing or understanding local cultural, spiritual and social dimensions of death and dying	● All humanitarians should reflect on their own cultural values, and engage with humility and respect ● Consult and collaborate with translators, local health professionals, and lay care providers to provide culturally and religiously sensitive palliative care
Equity	Providing palliative care to displaced persons may lead to concerns for equity if this care is not available to host communities	● Work with local communities to better understand and address their concerns ● Design programs in ways that explicitly address issues of equitable access to care ● Draw attention to the equity concerns of not providing palliation to those who require it, whether from refugee or host communities.
Continuity of care	Even if humanitarians initiated palliative care, continuity would be difficult if this approach does not exist in the local health system	● Thoroughly explore and support existing local palliative care provision ● Contribute to capacity building where needed, including training local lay people and health providers
Security	In some settings, security concerns may arise if when health professionals propose palliative care for a patient it is perceived by others as not providing the best care possible.	● Carefully and continuously assess security risks ● Ensure that health professionals are trained to evaluate such issues ● Explore ways as a team to still provide palliative care while not undermining team safety
Terminology	Some humanitarian and local health professionals and policymakers may resist the term ‘palliative care’ but be open to the clinical approach if not labeled in this way	● Consider how terms are understood and interpreted by different groups ● Seek to clarify meanings and adapt vocabulary used to the particular context

### Strengths and limitations

Limited empirical research has been conducted about palliative care in humanitarian settings [9]. This exploratory study helps to illuminate this important, but still under researched area of inquiry at a time when this topic is receiving increased attention in the humanitarian sector. Several limitations to our purposive sampling are also important to highlight. Despite our goals of diversity within our sample, we recruited more physicians (16) than other health professionals (7), and more individuals from high- (20) than low or middle-income countries (4). Additional perspectives from low and middle income countries have been gathered through the in-depth field studies that we have pursued as part of our broader research program. We would also characterize the individuals we spoke with as generally supporters of increasing palliative care approaches in humanitarian healthcare. While we sought to recruit participants who were more skeptical or critical, it proved difficult to do so.

## Conclusion

We have presented and discussed key obstacles to integrating palliative care approaches in humanitarian healthcare contexts. These range from more philosophical considerations to pragmatic and legal ones. Many of these obstacles have been identified by commentators, including the life-saving focus of humanitarian action, priority setting, lack of training, and insufficient policies or standards [5–7]. Our findings here provide further evidence regarding how these obstacles are perceived by humanitarian workers and policy-makers. Other obstacles have received little attention to date, including obstacles related to fundraising and security. Responding to these ten obstacles will require sustained attention, and a range of actions and interventions, both within and beyond humanitarian organizations. Further research on this topic is also warranted, especially to better understand obstacles to palliative care from the perspective of patients, care providers, and other stakeholders in locales affected by humanitarian crises. In Table 1, we summarize the obstacles and opportunities for how they are being or could be addressed – drawing both from the participants in our study and from the broader literature.

It is relevant in discussing the obstacles to palliative care in humanitarian contexts to also acknowledge a caution raised by our participants. While emphasizing the importance of identifying and overcoming obstacles to the provision of palliative care in humanitarian crises, several participants expressed that the possibility of providing palliative care must not be used to avoid providing curative care when doing so is feasible. Smith and Aloudat express a similar idea when they encourage humanitarians to adopt a dual commitment to “challenge suboptimal access to curative treatment where available, and the promotion of palliative care where appropriate.” [5] This dual commitment should be understood as essential for a coherent approach to overcoming obstacles to the integration of palliative and curative care in humanitarian healthcare.

Not addressing palliative care needs has enormous consequences for suffering and dying patients and their families. It may also have negative psychological consequences for healthcare providers [21, 24, 25] or harm the credibility of the organization [25]. Humanitarian organizations strive to provide equitable care and insist on optimizing the standard of care that is possible, even in very difficult conditions. Achieving these goals requires that humanitarian healthcare should seek to meet these tragedies with adequate resources for all affected, not to accept a lesser standard of care for some, particularly those who are the most vulnerable. Similar considerations are also being identified in the context of the COVID-19 pandemic, with calls to take steps to ensure that effective palliative care is optimized [26].

Since our gathering of these participants’ accounts, the WHO has made explicit recommendations for the integration of palliative care and symptom relief in humanitarian emergencies [12]. While the extent to which these recommendations have been adopted in practice remains uncertain, one feature is that they recast palliative care as integral to healthcare delivery for patients in a range of triage categories, beyond the category of expectant. Thus, for example, these include the recommendation that palliative care “be integrated with life-sustaining treatment as much as possible” ([12], p 16). For those who require treatment but are not in immediate danger of death, it notes that palliative care and/or symptom relief may be needed. These changes underscore the importance of palliative care and symptom relief for a range of patients, and for attending to the palliative needs of individuals for whom survival is not possible.

Humanitarian health care will necessarily involve encounters with persons for whom curative care is insufficient, or even impossible. Integrating palliative care as an essential part of humanitarian healthcare is vital in order to ensure that humanitarians fulfill their mandates and commitment not only to attempt to save lives, but also to alleviate suffering and promote human dignity, even in those instances when lives cannot be saved.

## Data Availability

The data analyzed during the current study are not publicly available due to confidentiality considerations. Additional illustrative quotations (excerpts) from the transcripts may be requested from the corresponding author.

## Abbreviations

NGO: Non-governmental organization
WHO: World Health Organization
